# CD4^+^CD28^null^ T Lymphocytes are Associated with the Development of Atrial Fibrillation after Elective Cardiac Surgery

**DOI:** 10.1038/s41598-018-28046-0

**Published:** 2018-06-25

**Authors:** Patrick Sulzgruber, Barbara Thaler, Lorenz Koller, Johanna Baumgartner, Arnold Pilz, Matthias Steininger, Sebastian Schnaubelt, Tatjana Fleck, Günther Laufer, Barbara Steinlechner, Max-Paul Winter, Georg Goliasch, Johann Wojta, Alexander Niessner

**Affiliations:** 1 Division of Cardiology, Department of Internal Medicine II, Medical University of Vienna, Vienna, Austria; 2 Division of Cardiac Surgery, Department of Surgery, Medical University of Vienna, Vienna, Austria; 3 Department of Anesthesia, General Intensive Care and Pain Management, Medical University of Vienna, Vienna, Austria; 4 Ludwig Boltzmann Cluster for Cardiovascular Research, Vienna, Austria

## Abstract

Post-operative atrial fibrillation (POAF) is postulated as a complex interaction of different pathogenic factors, suggesting inflammatory processes as a main trigger of this particular type of atrial fibrillation. Therefore, the study sought to assess the impact of cellular immunity on the development of POAF. Comparing patients developing POAF to individuals free of POAF the fraction of CD4^+^CD28^null^ T Lymphocytes was significantly higher in individuals developing POAF (11.1% [POAF] vs. 1.9% [non-POAF]; p < 0.001). CD4^+^CD28^null^ cells were independently associated with the development of POAF with an adjusted odds ratio per one standard deviation of 4.89 (95% CI: 2.68–8.97; p < 0.001). Compared to N-terminal Pro-Brain Natriuretic Peptide, the fraction of CD4^+^CD28^null^ cells demonstrated an increased discriminatory power for the development of POAF (NRI: 87.9%, p < 0.001; IDI: 30.9%, p < 0.001). Interestingly, a pre-operative statin-therapy was associated with a lower fraction of CD4^+^CD28^null^ cells (p < 0.001) and showed an inverse association with POAF (p < 0.001). CD4^+^CD28^null^ cells proved to be predictive for the development of POAF after cardiac surgery. Our results potentially indicate an auto-immune impact of this preexisting, highly cytotoxic T cell subset in the pathogenesis of POAF, which might be modified via the anti-inflammatory potential of a pre-operative statin-therapy.

## Introduction

Already in 2010 the total estimated burden of atrial fibrillation (AF) amounted to 33.5 million affected individuals worldwide, representing to most common cardiac arrhythmia in the western society^1^. While various types of AF are known in clinical practice posing a well investigated field in cardiovascular research, a specific type of AF occurring after cardiovascular surgery is still incompletely understood^2^. This so called post-operative atrial fibrillation (POAF), represents a common complication after surgical intervention with a reported incidence ranging from 15 to more than 45%^3^. POAF is associated with an increased length of hospitalization, a higher rate of procedure related complications, as well as poor patient outcome^4–6^. However, exact pathophysiological mechanisms that could explain the development and progression of POAF in this especially vulnerable patient collective are still scarce.

Recent evidence found a strong association of complement system activation and pro-inflammatory cytokines, mainly released by lymphocytes, with the onset of POAF – hinting to a potential impact of cellular immunity on the cardiac conduction system and in particular atrial fibrosis^7,8^.

CD4^+^ T lymphocytes lacking the surface-protein CD28 are crucially involved in inflammatory processes. While these so-called CD4^+^CD28^null^ T cells are rarely expressed in healthy individuals, their fraction strongly expands during the course of chronic inflammation. However, a co-stimulatory CD28-receptor on CD4 T cells is needed for cell-proliferation, regulation, activation, as well as cell-survival^9^. Based on longstanding inflammatory conditions, CD4^+^CD28^+^ T lymphocytes lose their expression of the CD28 antigen and therefore their sensitivity for both suppression and apoptosis induction by regulatory T cells, leading to an unimpaired inflammatory response as well auto-reactivity against human tissue^10,11^.

Recent evidence suggested a major impact of this T cell subset on the development of AF as well as on the prognosis of patients^12,13^. Considering recent findings of our study group, as well as previous observations on inflammation and the onset of AF, an analysis of the association of this specific T cell subset and the development of POAF might enlighten important prognostic as well as pathophysiologic insights into this dreading arrhythmia. Therefore, the present study aimed to investigate the impact of cytotoxic CD4^+^CD28^null^ T cells on the development of POAF after elective cardiac valve and/or coronary-artery bypass graft surgery.

## Methods

### Study Population

Within this prospective cohort study, we enrolled 129 patients undergoing elective cardiac valve and/or coronary-artery bypass graft (GABG) surgery including minimal invasive procedures. Patients were enrolled one day prior to surgical intervention at the Division of Cardiac Surgery, Department of Surgery of the Medical University of Vienna – a university affiliated tertiary care center. Enrollment was conducted between 08/2015 and 09/2016. Peripheral venous blood samples of all participants were available for further fluorescein-activated cell sorting (FACS) analysis and taken at the time of study enrollment. Furthermore, to ascertain the onset of an episode of AF after surgery, all participants received a permanent 6-lead surface ECG monitoring until discharge. Electronic ECG tracings of all individuals were continuously screened. AF episodes were documented and validated via a 12-lead surface ECG. POAF was defined in accordance to the guidelines of the European Society of Cardiology as a new onset of atrial fibrillating impulses (usually self-terminating) after major cardiac surgery in patients that were in sinus rhythm before surgical intervention^14^.

All participants gave written informed consent for study participation. For inclusion patients had to be free of any type of AF at the time of study enrollment and six months prior to the surgical intervention. However, all enrolled participants were AF naïve individuals, free of any remote history of AF. The medical records of all patients were screened for any kind of chronic inflammatory conditions, autoimmune disease, active infections or malignancies. Additionally, at the time of enrollment current values of inflammatory markers, such as C-reactive Protein or total Leucocyte count were screened for any major elevations. Patients presenting with any kind of inflammatory conditions, autoimmune disease, active infections or malignancies were not applicable for inclusion. The study protocol complies with the Declaration of Helsinki and was approved by the local ethics committee of the Medical University of Vienna (No. 1110/2013). Data reporting was performed according to the STROBE and MOOSE guidelines. Based on the prospective character of the study, there was no missing data in any of the tested variables. Range and consistent checks were regularly performed during the entire observation period.

### Data Acquisition and Flow Cytometry

Patient characteristics and routine laboratory parameters were assessed at the time of study inclusion/hospital admission and inserted into a pre-defined record abstraction form. Standardized assessment of post-operative laboratory values was conducted within 24 hours after the surgical intervention at the intensive care unit. Values of C-reactive protein (CRP) were screened daily during the postoperative hospitalization in order to elucidate the baseline level and the maximum increase after surgery in accordance to the local laboratory standards of the Medical University of Vienna (Cobas C System CHE2—Roche Diagnostics, Switzerland). Peripheral venous blood samples of all participants were taken at the time of study enrollment. Routine laboratory parameters were analyzed and processed according to local laboratory standards. Fresh heparinized EDTA blood samples were processed for further lymphocyte separation using a standardized protocol. For separation of lymphocytes and peripheral mononuclear cells from human whole blood – based on their buoyant density – a density gradient centrifugation was performed using a high-grade polyethylene barrier for density gradient separation. The lymphocyte layer after centrifugation was harvested and washed and furthermore mixed with a cryopreservation medium (Synth-a-Freeze Cyropreservation Medium, Thermo Fischer, Waltham, MA, USA) for storage at −80 degrees Celsius. After unfreezing and cell washing procedures using warmed PBS, cells were stained using PE conjugated Anti-CD4 (Biolegend, San Diego, CA, USA), as well as APC conjugated Anti-CD28 (Biolegend, San Diego, CA, USA) and incubated for 30 minutes. Regulatory T cells were identified via their intracellular forkhead-box protein P3 (Fox-P3) and CD25 expression using PE conjugated Anti-Fox-P3 (Biolegend, San Diego, CA, USA) as well Alexa-647 conjugated Anti-CD25 (Biolegend, San Diego, CA, USA) in a second FACS-panel using a permeabilization buffer for intercellular staining approach. For further analysis of stained cells BD FASC Canto II flow cytometry and FACSDiva were used. We assessed CD4^+^CD28^null^ cells after gating for total lymphocyte count, CD4 and CD28. CD4 cells are presented as percentage of lymphocytes. CD4^+^CD28^null^ cells are presented as percentage of CD4 cells.

### Statistical Analysis

Categorical data are illustrated as counts and percentages, continuous data are presented as median and interquartile range (IQR). Continuous variables were compared between groups using Mann-Whitey-U test. To assess differences of categorical data Chi-square test was used. Sperman-Rho correlation coefficient was used to elucidate the association of CD4^+^CD28^null^ frequencies and continuous variables. Binary logistic regression analysis was used to assess the influence of T cell subsets on the development of POAF. Continuous variables were log-transformed prior to their analysis in the regression model, to adjust data more closely to normal distribution. Data were presented as odds ratio (OR) and the respective 95% confidential interval (CI) per one standard deviation (1-SD) for continuous variables. The multivariate model 1 was adjusted for potential patient-specific confounders due to their association with the development of POAF: age, gender, previous myocardial infarction, valvular heart disease, hypertension, diabetes mellitus type 2, chronic obstructive pulmonary disease, chronic kidney disease, chronic heart failure. The multivariate model 2 was adjusted for potential peri- and post-operative confounders due to their association with the development of POAF: pre-operative statin therapy, LV volume index type of surgery, bicaval cannulation, time of cardiopulmonary bypass, aortic cross-clamp time and prolonged inotropic drug use. Classification and regression tree (CART) analysis was performed to elucidate cut-off values for CD4^+^CD28^null^ cell frequencies. An improvement in individual risk stratification of the fraction of CD4^+^CD28^null^ cells compared to NT-proBNP was examined using category-free net reclassification index (NRI) and discriminatory improvement index (IDI).

A two-sided p-value < 0.05 was defined as statistical significance. Statistical analyses were performed using the STATA 11 software package (StataCorp LP, USA) and SPSS 21.0 (IBM SPSS, USA).

### Data availability

The datasets generated and analyzed during the current study are available from the corresponding author on reasonable request.

## Results

### Baseline characteristics

After cardiac surgery, a total of 60 (46.5%) individuals out of 129 enrolled patients developed POAF, with a median time until the first onset of 2 days (IQR: 2–3). Detailed baseline characteristics for distribution of conventional risk factors and laboratory parameters stratified in patients developing POAF and individuals free of POAF are shown in Supplementary Table 1.

In brief, in the entire study collective the median age was 68 years and 72.3% of patients were male (n = 94). Moreover, CABG surgery was the most performed surgical intervention in our study collective (44.9%; n = 58) followed by single valve replacement (34.1%; n = 44) and combined GABG+ valve replacement surgery (20.9%; n = 27). Interestingly there was no difference in peri- and post-operative characteristics comparing individuals developing POAF to patients free of POAF.

With respect to cardiovascular comorbidities, we found comparable frequencies of hypertension (88.3% [POAF] vs. 87.0% [Non POAF]; p = 0.813), previous myocardial infarction (20.0% [POAF] vs. 31.9% [Non POAF]; p = 0.126), valvular heart disease (73.3% male [POAF] vs. 65.2% [Non POAF]; p = 0.517) and chronic heart failure (73.3% [POAF] vs. 65.2% [Non POAF]; p = 0.320). Interestingly, patients developing POAF tended to be more likely to present with diabetes mellitus type 2 (41.7% [POAF] vs. 27.5% [Non POAF]; p = 0.091) and chronic kidney disease (28.3% [POAF] vs. 15.9% [Non POAF]; p = 0.089) compared to individuals free of POAF.

### Distribution of T cell subsets

**Figure 1:**
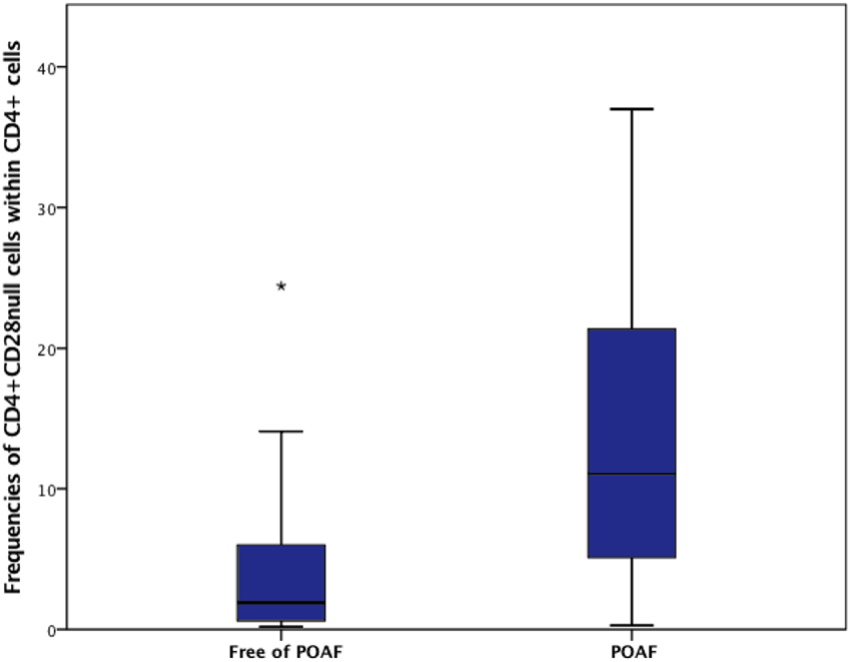
Boxplots showing frequencies of CD4^+^CD28^null^ cells within CD4^+^ cells comparing Patients free of POAF and individuals developing POAF (p < 0.001).

The fraction of CD4^+^CD28^null^ cells within CD4^+^ cells was significantly higher in patients developing POAF compared to their non-POAF counterparts (11.1% [POAF] vs. 1.9% [Non POAF]; p < 0.001) (Central Illustration). In contrast the fraction of both CD4^+^ cells within lymphocytes (38.2% [POAF] vs. 42.1% [Non POAF]; p = 0.492) and regulatory T cells within CD4^+^ cells (3.6% [POAF] vs. 3.5% [Non POAF]; p = 0.850) were comparable in patients developing POAF and individuals free of POAF (Table 1 and Fig. 1).

**Table 1:** Distribution of T cell subsets.

	POAF	Non POAF	p-value
Total lymphocytes, (IQR)	15828 (11762–22649)	17005 (12663–20825)	0.804
% CD4^+^ cells within lymphocytes, (IQR)	38.2 (33.1–46.9)	42.1 (31.6–48.7)	0.492
% CD4^+^CD28^null^ cells within CD4^+^ cells, (IQR)	11.1 (4.8–21.4)	1.9 (0.6–6.2)	**<0.001**
% Regulatory T cells within CD4^+^ cells, (IQR)	3.6 (2.2–4.7)	3.5 (2.0–4.9)	0.850

Continuous data are presented as median (interquartile range) and were compared between subgroups using Mann-Whitney-U test.

After stratification in tertiles of CD4^+^CD28^null^ T cell frequencies, we found an increasing incidence of POAF with increasing CD4^+^CD28^null^ T cell frequencies (p < 0.001). Interestingly, we observed that frequencies of CD4^+^CD28^null^ T cells showed a moderate correlation with both maximum CRP values after surgery (r = 0.214; p = 0.020) and a marginally significant correlation with the total leucocyte count after surgery (r = 0.185; p = 0.046) (Table 2).

**Table 2:** Baseline Characteristics stratified by Tertiles of CD4^+^ CD28^null^ Cell Frequencies.

		Tertile 1	Tertile 2	Tertile 3	p-value	r=	*p-value
% CD4^+^CD28^null^ cells within CD4+ cells, (IQR)		0.7 (0.4–1.4)	5.5 (3.7–7.1)	17.6 (12.1–25.2)	**<0.001**		
POAF, n (%)		6 (14.0)	18 (41.9)	36 (83.7)	**<0.001**		
First onset after Surgery, days (IQR)		2 (2–3)	2 (2–3)	2 (2–3)	0.247	0.013	0.922
**Clinical Presentation**							
Age, years (IQR)		69 (54–75)	66 (59–73)	68 (61–74)	0.599	0.028	0.756
Male gender, n (%)		34 (79.1)	28 (65.1)	32 (74.4)	0.334		
Current Smoker, n (%)		4 (9.3)	5 (11.6)	1 (2.3)	0.244		
*Type of Surgery*	Valve Replacement, n (%)	8 (18.6)	18 (41.9)	18 (41.9)	0.055		
	CABG, n (%)	25 (58.1)	19 (44.1)	14 (32.6)			
	Valve Replacement and CABG, n (%)	10 (23.3)	6 (14.0)	11 (25.5)			
*Location of Valve Surgery*	Aortic Valve, n (%)	5 (27.8)	9 (37.5)	6 (20.7)	0.510		
	Mitral Valve, n (%)	3 (16.7)	4 (16.7)	9 (31.0)			
	Tricuspid Valve, n (%)	0 (−)	1 (4.2)	0 (−)			
	Combined, n (%)	10 (55.6)	10 (41.7)	14 (48.3)			
LA volume index, mL/m^2^ (IQR)		45.8 (38.9–55.0)	45.6 (38.9–58.6)	45.8 (31.7–61.1)	0.802	0.058	0.513
**Comorbidities**							
Previous MCI, n (%)		9 (20.9)	16 (37.2)	9 (20.9)	0.141		
Family History in AF, n (%)		24 (55.9)	25 (58.1)	24 (55.8)	0.969		
Valvular Heart Disease, n (%)		34 (79.1)	29 (67.4)	28 (65.1)	0.315		
Hypertension, n (%)		36 (83.7)	26 (83.7)	41 (95.3)	0.168		
Diabetes Mellitus Type II, n (%)		7 (16.3)	17 (39.5)	20 (46.5)	**0.008**		
Chronic Kidney Disease, n (%)		6 (14.0)	11 (25.6)	11 (25.6)	0.320		
Peripheral Vascular Disease, n (%)		3 (6.9)	4 (9.3)	4 (9.3)	0.905		
COPD, n (%)		0 (−)	1 (2.3)	0 (−)	0.365		
Chronic Heart Failure, n (%)		30 (69.8)	28 (65.1)	31 (72.1)	0.776		
*NYHA-Class, n (%)*	II	16 (53.3)	19 (67.9)	17 (54.8)	0.543		
	III	13 (43.3)	9 (32.1)	13 (41.9)			
	IV	1 (3.3)	0 (−)	0 (−)			
**Perioperative Management**							
Aortic cross-clamp time, min (IQR)		72 (63–81)	66 (63–73)	69 (66–74)	0.253	0.038	0.673
CPB time, min (IQR)		109 (90–133)	95 (90–110)	118 (101–128)	**0.022**	0.085	0.339
Bicaval cannulation, n (%)		2 (4.7)	4 (9.3)	3 (6.9)	0.699		
Intraoperative complication, n (%)		1 (2.3)	0 (−)	0 (−)	0.365		
Intraoperative inotropic use, n (%)		18 (41.9)	17 (39.5)	20 (46.5)	0.801		
**Postoperative Management**							
Prolonged Inotropic drug use >72 h, n (%)		1 (2.3)	2 (4.7)	1 (2.3)	0.773		
RBC Transfusion, n (%)		17 (39.5)	23 (53.5)	15 (34.9)	0.192		
Sepsis, n (%)		1 (2.3)	2 (4.7)	0 (−)	0.359		
Cardiogenic Shock, n (%)		1 (2.3)	1 (2.3)	1 (2.3)	1.000		
Major Bleeding, n (%)		0 (−)	0 (−)	1 (2.3)	0.365		
Surgical Revision, n (%)		0 (−)	1 (2.3)	0 (−)	0.365		
**Laboratory Measures**							
Creatinine at admission, mg/dl (IQR)		0.91 (0.74–1.06)	0.96 (0.83–1.37	0.97 (0.86–1.30)	**0.036**	0.149	0.094
Cholesterol at admission, mg/dl (IQR)		176 (141–204)	163 (133–194)	170 (136–193)	0.482	−0.093	0.301
ALT at admission, U/l (IQR)		21 (17–33)	25 (17–31)	24 (17–36)	0.641	0.109	0.222
AST at admission, U/l (IQR)		22 (18–30)	22 (17–28)	23 (17–27)	0.986	0.050	0.576
Gamma-GT at admission, U/l (IQR)		30 (16–56)	27 (20–34)	25 (20–52)	0.660	−0.068	0.446
TSH at admission, yU/l (IQR)		1.39 (0.82–1.85)	1.30 (0.81–1.98)	1.25 (0.79–2.10)	0.952	0.033	0.721
NT-proBNP at admission pg/ml (IQR)		416.4 (177.7–915.6)	461.4 (153.0–1751.0)	678.1 (248.0–1758.0)	0.154	0.146	0.102
NT-proBNP after surgery, pg/ml (IQR)		2096.0 (532.4–3173.5)	2184.0 (1039.0–8682.0)	2645.5 (1648.0–4983.2)	0.529	0.176	0.249
CRP before surgery, mg/dl (IQR)		0.21 (0.08–0.38)	0.15 (0.08–0.34)	0.15 (0.8–0.32)	0.852	−0.077	0.392
CRP max. after surgery, mg/dl (IQR)		14.0 (8.9–21.8)	19.3 (13.8–23.5)	20.9 (15.7–24.2)	0.087	0.214	**0.020**
Leucocytes after surgery, thousand/µl		14.5 (12.5–17.1)	14.5 (11.2–20.1)	14.6 (12.9–21.0)	0.334	0.185	**0.046**
Lactate after surgery, mmol/l (IQR)		2.4 (1.6–2.9)	2.2 (1.6–2.8)	2.4 (1.7–3.1)	0.708	−0.022	0.822
**Medication at admission**							
ASA, n (%)		35 (81.4)	27 (62.7)	33 (76.7)	0.125		
Other NSAID, n (%)		2 (4.7)	1 (2.3)	0 (−)	0.359		
ACE Inhibitor, n (%)		17 (39.5)	16 (37.2)	16 (37.2)	0.968		
Beta Blockers, n (%)		24 (55.8)	25 (58.1)	29 (67.4)	0.506		
Statins, n (%)		34 (79.1)	21 (48.8)	16 (37.2)	**<0.001**		

Categorical data are presented as counts and percentages, continuous as median and IQR (interquartile range). Categorical data are analyzed using Chi-square-test, continuous data using Kruskal-Wallis test. Association of continuous variables was assessed by Sperman-Rho correlation coefficient. *p-value for correlation. AF = Atrial Fibrillation, POAF = Post-Operative Atrial Fibrillation, CABG = Coronary Artery Bypass Graft, COPD = Chronic Obstructive Pulmonary Disease, CPB = Cardio-Pulmonary Bypass, RBC = Red Blood Cell, ALT = Alanine Transaminase, AST = Aspartat Transaminase, BNP = Brain Natriuretic Peptide, CRP = C-Reactive Protein, TSH = Thyroid-stimulating Hormone, ASA = Acetylsalicylic Acid, NSAID = Non-Steroid Anti-Inflammatory Drug, ACE = Angiotensin Converting Enzyme.

Of utmost interest, patients receiving a lipid-lowering therapy using statins were found to have lower median frequencies of CD4^+^CD28^null^ cells (7.4% [no statins] vs. 2.7% [statins]; p < 0.001), compared to non-recipients.

### Binary logistic regression analysis

The fraction of CD4^+^CD28^null^ cells within CD4^+^ cells was significantly associated with the development of POAF with a crude OR per 1-SD of 4.34 (95% CI: 2.53–7.47; p < 0.001). Both the fraction of CD4^+^ cells within lymphocytes (crude OR per 1-SD of 0.93 [95%CI: 0.65–1.32; p = 0.689]) and regulatory T cells within CD4+ cells (crude OR per 1-SD of 1.04 [95%CI: 0.74–1.48; p = 0.802]) did not show a predictive potential for the development of POAF. Even after adjustment for a large variety of potential confounders in the multivariate model 1, the fraction of CD4^+^CD28^null^ cells remained strong and independently associated with the development of POAF with an adjusted OR per 1-SD of 4.89 (95% CI: 2.68–8.97; p < 0.001). Similar results applied after adjustment in the multivariate model 2 with an adjusted OR per 1-SD of 6.02 (95% CI: 2.98–12.07; p < 0.001) (see Table 3). Moreover, the predictive potential of CD4^+^CD28^null^ cell frequencies on the development of POAF was irrespective of the performed type of surgery, mirroring its applicability in all performed subtypes of surgical interventions (CABG: 2.69 [1.57–4.61], p < 0.001; Valve: 5.35 [1.82–15.70], p = 0.002; GABG+ Valve: 2.57 [1.21–5.43], p = 0.014). Additionally, a statin therapy prior to surgical intervention showed an inverse association with the development of POAF with a crude OR of 0.27 (95%CI: 0.13–0.57; p < 0.001). Moreover, interaction term analysis showed a modification of the predictive effect of the fraction of CD4^+^CD28^null^ cells by pre-operative statin-therapy (p = 0.055).

**Table 3:** Binary Logistic Regression Analysis.

	Crude OR (95% CI)	p-value		*Adj. OR (95% CI)	p-value
% CD4^+^ cells within lymphocytes	0.93 (0.65–1.32)	0.689	Model 1*	0.89 (0.61–1.31)	0.695
			Model 2**	0.91 (0.61–1.33)	0.618
% CD4^+^CD28^null^ cells within CD4^+^ cells	4.34 (2.53–7.47)	**<0.001**	Model 1*	4.89 (2.68–8.97)	**<0.001**
			Model 2**	6.02 (2.98–12.07)	**<0.001**
% Regulatory T cells within CD4^+^ cells	1.04 (0.74–1.48)	0.802	Model 1*	0.93 (0.63–1.38)	0.739
			Model 2**	1.13 (0.77–1.67)	0.525

Binary logistic regression model for the association of T cell subsets on the development of POAF. Odds ratios (OR) for continuous variables refer to a 1-SD increase.

*The multivariate model 1 was adjusted: age, gender, previous myocardial infarction, valvular heart disease, hypertension, diabetes mellitus type 2, chronic obstructive pulmonary disease, chronic kidney disease, chronic heart failure.

**The multivariate model 2 was adjusted: pre-operative statin therapy, LV volume index, type of surgery, bicaval cannulation, time of cardiopulmonary bypass, aortic cross-clamp time and prolonged inotropic drug use.

**Figure 2:**
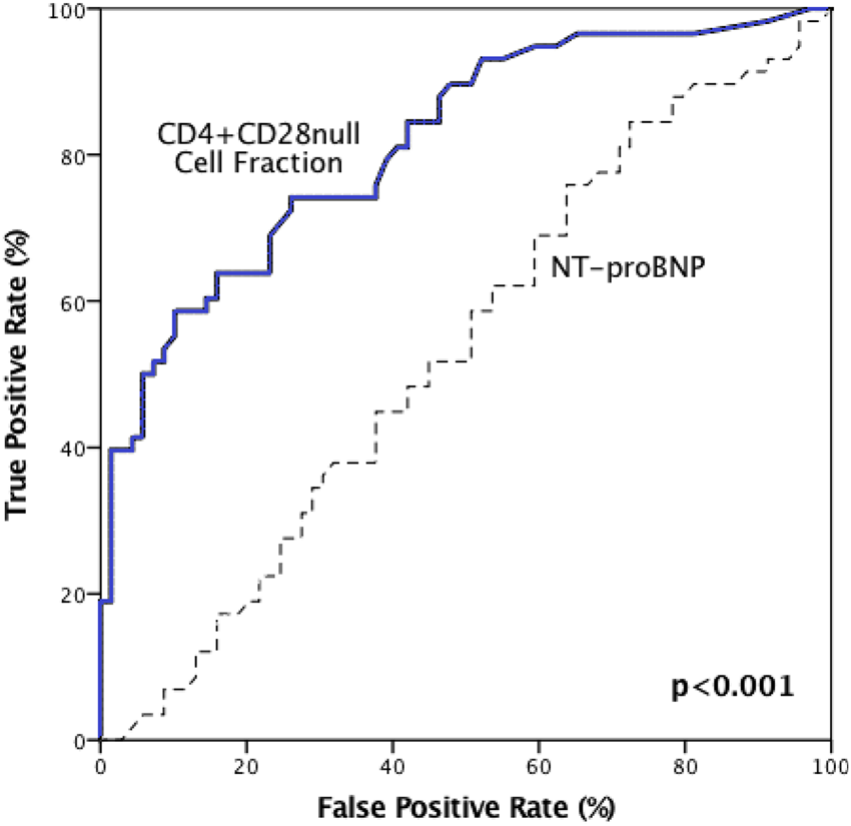
Receiver operating characteristics (ROC) curve comparing the discriminatory power of frequencies of CD4^+^CD28^null^ Cells (AUC: 0.821) and NT-proBNP values (AUC: 0.533) for the development of POAF (p < 0.001).

The area under the curve (AUC) for the fraction of CD4^+^CD28^null^ cells on the development of POAF was good with 0.821, while the AUC for NT-proBNP proved to be lower with 0.533 (p < 0.001) (see Fig. 2).

Using CART-Analysis cut-off values for the prediction of POAF were defined as CD4^+^CD28^null^ frequencies of 15.0% (=upper value) to 4.0% (=lower value) showing a sensitivity of 80.0% and a specificity of 77.8%. The positive predictive value was 64.0%, the negative predictive value 60.9% and the accuracy 69.7%.

The fraction of CD4^+^CD28^null^ cells added prognostic value beyond the multivariate model compared to NT-proBNP as the strongest predictor for POAF described in literature, indicated by improvements in both the category-free net reclassification index (NRI: 87.9%; p < 0.001) and the discriminatory improvement index (IDI: 30.9%; p < 0.001)^15^.

## Discussion

In the current study, we evaluated the association of CD4^+^CD28^null^ cells with the development of POAF in patients undergoing elective cardiac valve and/or CABG surgery. The presented data are the first in literature describing an impact of cellular immunity – more specifically the CD4^+^CD28^null^ T cells subset – on development of POAF.

### Inflammation in Cardiovascular Disease and Atrial Fibrillation

Both structural heart disease and AF proved to be closely linked to inflammation, inflammatory cytokines and cellular immunity^12,13,16–19^. Based on recent findings, that the CD4^+^CD28^null^ T cell subset is associated with AF in chronic cardiac inflammation, we aimed to examine its association with the first onset of POAF after surgical conditions, representing a strong stimulus for acute cardiac inflammation. This terminally differentiated T cell line is characterized by the release of interferon-gamma (IFN-gamma), tumor necrosis factor-alpha (TNF-α), and most interestingly interleukin-2 (IL-2)^17^. Of note, recent evidence revealed that both elevated IL-2 and TNF-α levels were directly associated with an increased risk for the development of POAF^18–20^. Therefore, those observations seem to be in line with our findings showing a significantly higher fraction of CD4^+^CD28^null^ T cells within CD4+ cells among patients developing POAF compared to patients free of POAF^18–24^.

Moreover, CRP was found as a strong predictor for the development of AF in a population based cohort study by Aviles and colleagues^25^. These finding have recently been confirmed in individuals undergoing cardiovascular surgery, showing that elevated CRP values after surgery were independently associated with the onset of POAF^7^. Those data are similar to our results, showing elevated maximum CRP values after surgery in patients developing POAF compared to their non-POAF counterparts. The observed correlation of maximum CPR values and frequencies of CD4^+^CD28^null^ T cell might mirror an aggravated inflammatory response after surgery – predominantly triggered by a pre-existing elevated CD4^+^CD28^null^ cell fraction in those individuals. Since 21% of enrolled patient received a combined GABG and valve surgery, it seems intuitive that those individuals experienced extended myocardial tissue damage, leading to an aggravated inflammatory response favoring the development of POAF. Therefore, the high proportion of combined surgical procedures might also explain in addition to the increased patient age the observed high overall incidence (46.5%) of POAF in the present study population. In accordance to evidence in literature, we observed that patients undergoing combined surgical procedures (CABG+ valve; multiple valve) presented with a higher incidence of POAF than single interventions (combined: 56.3% vs. single: 40.7%). Similarly, maximum CRP values proved to be elevated in the combined intervention subgroup (combined: 20.0 mg/dl [14.0–27.2] vs. single: 17.3 mg/dl [11.2–22.6]).

### CD4^+^CD28^null^ T cells and Cardiac Inflammation after Surgery

Based on continuous auto-antigen presentation and stimulation, CD4^+^CD28^null^ T cells showed cell-expansion and – of utmost importance – auto reactive behavior against healthy human tissue^11,17,26,27^. In this context, due to the loss of the CD28^+^ surface antigen this T cell subset evolves on cytotoxic characteristics – similar to natural killer cells – producing cytolytic enzymes, such as perforin or granzyme A and B, which are known for their deleterious impact on both smooth vascular muscle cells and also myocardial tissue^10,28–31^. Due to an auto-aggressive behavior of this cytotoxic T cell subset, multiple micro-scars are formed based on cell-necrosis and apoptosis induction, leading to atrial fibrosis. As recently demonstrated, atrial fibrosis is known to impact on cardiac eclectic conduction, generating fibrillation impulses among atrial tissue and resulting in the manifestation of POAF^32^. Of note, the observed significant association of CD4^+^CD28^null^ cells and of both CRP and total leucocyte values in patients developing POAF underlines the aggravated inflammatory state in this subgroup, compared to non-POAF individuals. Therefore, an unimpaired local inflammatory response by CD4^+^CD28^null^ cells – triggered by surgical procedures and auto-antigen presentation – which results in an auto-aggressive and cytotoxic environment among myocardial tissue, needs to be assumed. Interestingly, comparing patients with high frequencies (>15%) of CD4^+^CD28^null^ cells that developed POAF to non-POAF individuals, we found lower maximum CRP values in non-POAF individuals (POAF: 21.71 mg/dl vs. non-POAF: 18.65 mg/dl). This observation fosters our assumption, that the inflammatory response of CD4^+^CD28^null^ cells was – despite high frequencies – lower in non-POAF individuals and therefore potentially posing a decreased odd for the onset of POAF.

Unfortunately, we were not able to find a significant correlation of frequencies of CD4^+^CD28^null^ cells and creatin-kinase (CK) values after surgery as a sensitive marker for myocardial tissue damage (data not shown). However, based on extensive tissue damage due to prior surgical intervention, the auto-reactive impact of CD4^+^CD28^null^ on myocardial tissue damage might be masked.

Of utmost interest, we found that the fraction of CD4^+^CD28^null^ cells was significantly lower in individuals receiving a lipid-lowering therapy using statins, compared to non-recipients. In this context, the benefit of a peri-operative statin therapy has already been described in literature in several randomized controlled trials, showing a reduced risk for the development of POAF^33^. Therefore, our findings imply that statins potentially lower the frequency of this cytotoxic T cell subset via its anti-inflammatory potential, modifying the post-operative inflammatory response and therefore avoiding the development of POAF^34^.

### Post-Operative Atrial Fibrillation – A Surgery-Triggered Autoimmune Disease?

The strong association of CD4^+^CD28^null^ cells and the development of POAF could be explained by the impact of myocardial tissue damage on cardiac electric conduction.

Of note in patients undergoing cardiac surgery, the fraction of this T cell subset has already been extended during longstanding cardiovascular disease (e.g. long-term hypertension, coronary vessel disease, myocardial infarction or cardiac valve disease) in stable conditions at baseline prior to the surgical intervention – the mechanic stimulus of the surgery per se seems to act as a trigger for CD4^+^CD28^null^ mediated inflammatory activation. The triggered auto-aggressive inflammatory response by this extensively over-expressed cytotoxic T cell subset potentially promotes micro-scar formation among myocardial tissue which is known to impact on cardiac electric conduction, leading to fibrillating impulses among vulnerable atrial tissue and resulting in the manifestation of POAF.

Considering the potential pathophysiological origin of this presumed immune-mediated type of AF, the time until first arrhythmic onset might differ to other etiologies of POAF. However, we observed a balanced time-to-onset between tertiles of CD4^+^CD28^null^ cells – which does not support this assumption. Taking into account that this sub-analysis might be underpowered based on the low number of POAF cases, this issue might deserve further attention in larger investigations on this topic.

The observed elevated NT-proBNP values in patients developing POAF potentially mirror an aggravated cardiac strain based on micro-scar formation among cardiac tissue. As a routinely available biomarker, NT-proBNP is known as a strong predictor for both major cardiac adverse events and the development of POAF after cardiac surgery^12^. Of note CD4^+^CD28^null^ cells at baseline showed an excellent discriminatory power, even demonstrating superiority to pre-operative NT-proBNP values as predictive marker. Therefore, baseline assessment of CD4^+^CD28^null^ cell frequencies prior to surgical intervention could be used for valid risk stratification for the development of POAF in patients undergoing cardiac surgery.

### Limitations

The major limitation of the current analysis represents its small sample size. However, taking into consideration that FACS analysis was applied for cell analysis, a satisfactory total number of participants has been enrolled.

## Conclusion

Higher frequencies of CD4^+^CD28^null^ T cells were found in patients developing POAF. We were able to demonstrate that CD4^+^CD28^null^ cells were strongly and independently associated with the development of POAF. Most importantly, while the prognostic value of CD4^+^CD28^null^ cells in CHF-patients with AF has been previously described, we extended the current knowledge by demonstrating that CD4^+^CD28^null^ cells are clearly associated with the first onset of POAF. Therefore, CD4^+^CD28^null^ cells might be assessed for risk stratification in the pre-operative management of patients prior to elective cardiac valve and/or CABG surgery. However, despite the fact, that there is currently no valid option for sufficient medical treatment on the suppression of CD4^+^CD28^null^ cell activity, it represents a potential therapeutic target for future investigations. In this context, patients receiving statin-therapy were found to have lower frequencies of CD4^+^CD28^null^ cells even showing a strong inverse association with development of POAF. Therefore, to avoid cardiac arrhythmia via down-regulation of the fraction of CD4^+^CD28^null^ cells, a safe and feasible pre-optative stain-therapy could represent a potential therapeutic agent.

## Electronic supplementary material

Supplementary Table 1
