# Supplementary material for: CD4+CD28null T Lymphocytes are Associated with the Development of Atrial Fibrillation after Elective Cardiac Surgery
Source: Sci Rep. 2018 Jun 25;8:9624. doi: 10.1038/s41598-018-28046-0 (PMC6018098; doi:10.1038/s41598-018-28046-0)
Supplement: Supplementary file 1 — Supplementary Table 1 [file 41598_2018_28046_MOESM1_ESM.pdf]

$CD4^{+}CD28^{null}$ T Lymphocytes are Associated with the Development of Atrial Fibrillation after Elective Cardiac Surgery

Patrick Sulzgruber, MD, PhD, MBA; Barbara Thaler, MSc; Lorenz Koller, MD; Johanna Baumgartner, BSc; Arnold Pilz, BA; Matthias Steininger, MD; Sebastian Schnaubelt; Tatjana Fleck, MD; Günther Laufer, MD; Barbara Steinlechner, MD; Max-Paul Winter, MD; Georg Goliasch, MD, PhD; Johann Wojta, PhD; Alexander Niessner, MD, MSc

Supplementary Table 1: Baseline Characteristics Comparing Patients Developing POAF and Non-POAF Individuals

	POAF (n=60)	Non POAF (n=69)	p-value
**Clinical Presentation**			
Age, years (IQR)	69 (61-74)	68 (57-74)	0.197
Male gender, n (%)	39 (65.0)	50 (79.7)	0.061
Current Smoker, n (%)	6 (10.0)	4 (5.8)	0.373
Type of Surgery			0.236
Valve Replacement, n (%)	21 (35.0)	23 (33.3)	
CABG, n (%)	23 (38.3)	35 (50.7)	
Valve Replacement and CABG, n (%)	16 (26.7)	11 (15.9)	
Location of Valve Surgery			0.718
Aortic Valve, n (%)	10 (27.0)	10 (29.4)	
Mitral Valve, n (%)	8 (21.6)	8 (23.5)	
Tricuspid Valve, n (%)	0 (-)	1 (2.9)	
Combined, n (%)	19 (55.9)	15 (44.1)	
LA volume index, mL/m^2 (IQR)	56.3 (36.1-65.6)	44.0 (34.5-51.4)	<0.001
**Comorbidities**			
Previous MCI, n (%)	12 (20.0)	22 (31.9)	0.126
Family History in AF, n (%)	33 (55.0)	40 (58.0)	0.406
Valvular Heart Disease, n (%)	44 (73.3)	47 (68.1)	0.517
Hypertension, n (%)	53 (88.3)	60 (87.0)	0.813
Diabetes Mellitus Type II, n (%)	25 (41.7)	19 (27.5)	0.091
Peripheral Vascular Disease, n (%)	6 (10.0)	5 (7.2)	0.753
COPD, n (%)	1 (1.6)	0 (-)	0.282
Chronic Kidney Disease, n (%)	17 (28.3)	11 (15.9)	0.089
Chronic Heart Failure, n (%)	44 (73.3)	45 (65.2)	0.320
NYHA-Class, n (%)			0.202
II	23 (52.3)	30 (66.7)	
III	21 (47.7)	15 (31.1)	
IV	0 (-)	1 (2.2)	
**Perioperative Management**			
Aortic cross-clamp time, min (IQR)	68 (65-78)	69 (63-78)	0.753
CPB time, min (IQR)	110 (90-124)	107 (91-130)	0.983
Bicaval cannulation, n (%)	5 (8.3)	4 (5.8)	0.209
Intraoperative complication, n (%)	1 (1.6)	0 (-)	0.282
Intraoperative inotropic use, n (%)	29 (48.3)	26 (37.7)	0.222
**Postoperative Management**			
Prolonged Inotropic drug use >72h, n (%)	3 (4.8)	1 (1.4)	0.246
RBC Transfusion, n (%)	34 (56.7)	21 (30.4)	**0.003**
Sepsis, n (%)	1 (1.6)	2 (2.8)	0.643
Cardiogenic Shock, n (%)	2 (3.2)	1 (1.4)	0.643
Major Bleeding, n (%)	1 (1.6)	0 (-)	0.282
Surgical Revision, n (%)	1 (1.6)	0 (-)	0.282
**Laboratory Measures**			
Creatinine at admission, mg/dl (IQR)	0.92 (0.83-1.31)	0.96 (0.79-1.17)	0.504
Cholesterol at admission, mg/dl (IQR)	165 (134-190)	176 (139-200)	0.181
ALT at admission, U/l (IQR)	23 (17-28)	22 (18-28)	0.831
AST at admission, U/l (IQR)	25 (18-35)	22 (17-33)	0.179
Gamma-GT at admission, U/l (IQR)	27 (19-51)	27 (17-41)	0.909
TSH at admission, yU/l (IQR)	1.28 (0.80-1.87)	1.39 (0.85-2.17)	0.425
NT-proBNP at admission pg/ml (IQR)	525 (233-1636)	481 (180-1651)	0.520
NT-proBNP after surgery, pg/ml (IQR)	2282 (1651-5164)	1878 (541-3749)	0.185
CRP before surgery, mg/dl (IQR)	0.11 (0.10-0.29)	0.21 (0.10-0.37)	0.115
CRP max. after surgery, mg/dl (IQR)	19.31 (14.06-23.96)	17.52 (9.82-22.74)	0.122
Lactate after surgery, mmol/l (IQR)	2.35 (1.70-2.70)	2.45 (1.60-3.20)	0.560
**Medication at admission**			
ASA, n (%)	48 (80.0)	47 (68.1)	0.126
Other NSAID, n (%)	1 (1.6)	2 (2.9)	0.643
ACE Inhibitor, n (%)	24 (40.0)	25 (36.2)	0.422
Beta Blockers, n (%)	40 (66.7)	38 (55.1)	0.644
Statins, n (%)	23 (38.3)	48 (69.6)	**<0.001**

Categorial data are presented as counts and percentages, continuous as median and IQR (interquartile range). Categorical data are analysed using Chi-square-test, continuous data using Mann-Whitney U test. AF=Atrial Fibrillation, POAF=Post-Operative Atrial Fibrillation, CABG=Coronary Artery Bypass Graft, COPD = Chronic Obstructive Pulmonary Disease, CPB = Cardio-Pulmonary Bypass, RBC = Red Blood Cell, ALT=Alanine Transaminase, AST=Aspartat Transaminase, BNP=Brain Natriuretic Peptide, CRP=C-Reactive Protein, TSH=Thyroid-stimulating Hormone, ASA = Acetylsalicylic Acid, NSAID = Non-Steroid Anti-Inflammatory Drug, ACE = Angiotensin Converting Enzyme
